# Maximal electrocatalytic activity of Sr-doped Nd_1−*x*_Sr_*x*_MnO_3_ perovskite oxides (*x* = 0.1, 0.2, 0.3) towards ORR: for next-generation power systems

**DOI:** 10.1039/d6ra00332j

**Published:** 2026-03-09

**Authors:** B. B. Nayak, R. R. Das, P. Parida, P. Parhi

**Affiliations:** a Department of Chemistry, Ravenshaw University Cuttack Odisha-753003 India pparhi@ravenshawuniversity.ac.in +91-8895193144

## Abstract

By using the sol–gel method, a series of Nd_1−*x*_Sr_*x*_MnO_3_ perovskite oxides (*x* = 0.1, 0.2, 0.3), designated as NSM-0.9, NSM-0.8, and NSM-0.7, were prepared and characterized using analytical techniques including XRD, FESEM, TEM, EDS, and XPS. Our investigation revealed that NSM-0.7 (Nd_0.7_Sr_0.3_MnO_3_) is the most effective electrocatalyst for the oxygen reduction reaction (ORR). Its superior electrocatalytic performance in a 0.1 M KOH solution, evaluated with RDE and RRDE techniques, was quantified by an onset potential (*E*_on_) of 0.82 V *vs.* RHE, a half-wave potential (*E*_1/2_) of 0.58 V *vs.* RHE, a limiting current density (*J*_L_) of −5 mA cm^−2^, which is the same as the current density of Pt/C, and a kinetic current density (*J*_k_) of 0.41 mA cm^−2^ at 1600 rpm. This material also favoured a highly efficient 4e^−^ pathway with the formation of a minimal amount of H_2_O_2_. NSM-0.7's superior catalytic performance is attributed to optimal Sr-doping at the perovskite's A-site, a process that significantly enhances its Mn valence and oxygen adsorption capacity. Furthermore, chronoamperometry confirmed that NSM-0.7 exhibits superior stability compared to the benchmark Pt/C catalyst, demonstrating that strategic A-site doping is a promising approach for improving conventional perovskite oxides for electrocatalytic applications.

## Abstract 2

Nd_1−*x*_Sr_*x*_MnO_3_ perovskite oxides (*x* = 0.1, 0.2, 0.3), designated as NSM-0.9, NSM-0.8, and NSM-0.7, were prepared by Sol–Gel method and their electrocatalytic properties towards ORR was evaluated.

## Graphical abstract

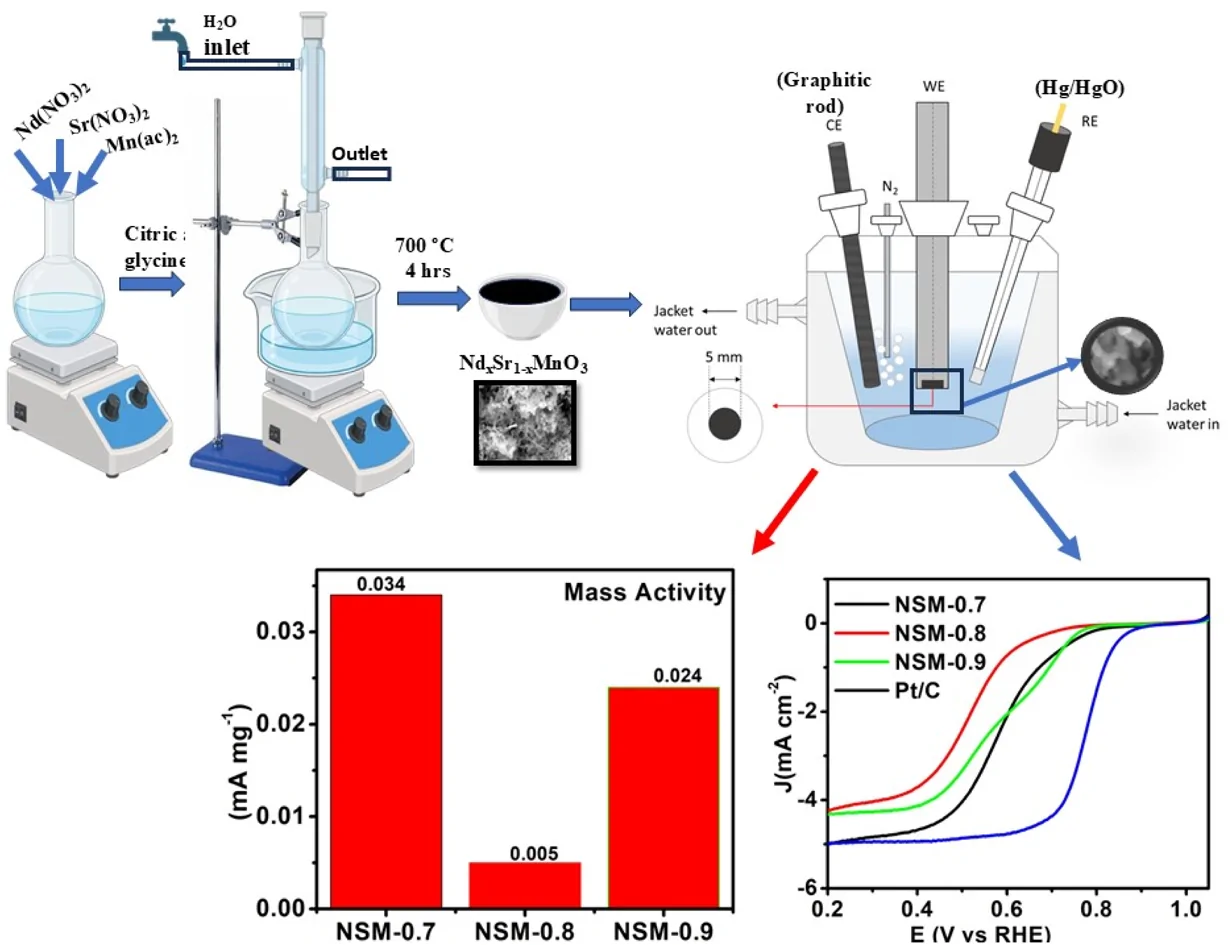

## Introduction

The demand for sustainable and eco-friendly energy has resulted in there being significant focus on various technologies related to energy transformation and accumulation.^1–4^ Fuel cells, supercapacitors, metal air batteries, *etc.*, have gained remarkable attention over the past few years.^5–7^ Among various fuel cells, Direct Methanol Fuel Cells (DMFCs) offer a promising path forward for power conversion technology, offering high energy density, efficiency, and low emissions as a solution to the inadequacy of non-renewable fossil fuels in meeting global energy demands and their contribution to environmental degradation.^8–10^ The main hurdle in the expansion of different fuel cells is the sluggish oxygen reduction reaction (ORR) kinetics of cathodic reactions. The ORR is a critical cathodic pathway that governs the power output of devices, making it essential for numerous renewable energy technologies, such as fuel cells and metal–air batteries.^11,12^ While platinum (Pt) is the benchmark ORR catalyst, its widespread application is hindered by its high cost, scarcity, and limited durability.^13^

Over the past few years, researchers have increasingly focused on perovskite oxides as a more affordable alternative to expensive noble metals for the ORR due to their remarkable structural and compositional stability.^14,15^ Perovskite oxides follow the general formula ABO_3_, featuring a crystal structure where the A-site cation (a rare-earth metal) sits at the corners, and the B-site cation (a transition metal) is at the centre, surrounded by six oxygen atoms.

Lanthanum-based perovskites have recently emerged as a promising, cost-effective alternative to expensive platinum (Pt)-based catalysts for the ORR due to their impressive electrocatalytic properties.^16–18^ A comprehensive review of various LnMnO_3_ (where Ln is a rare earth element) perovskites revealed that lanthanum-based variants demonstrated the highest ORR activity. The observed trend in electrocatalytic activity among these perovskites follows the order: Yb > DY > Y > Gd > Sm > Nd > Pr > La.^19^

A key advantage of perovskites is their tunable nature; by substituting A- or B-site atoms, their structure, oxygen content, and catalytic activity can be precisely tailored.^20,21^ For example, substituting strontium (Sr) at the A-site is a highly effective strategy for enhancing the material's conductivity, oxygen ion mobility, and catalytic ability to dissociate oxygen.^22^ Studies have shown that doping the A- and B-sites with specific elements can significantly improve catalytic activity. For example, Hyodo *et al.* demonstrated that La_0.4_Sr_0.6_MnO_3_ is an extremely effective electrocatalyst for non-aqueous Li–O_2_ batteries.^23^ Similarly, according to Li *et al.*, doping lanthanum manganite (LaMnO_3_) composites with calcium can tune their catalytic activity for zinc–air battery applications.^24^ Another effective strategy involves creating a deficiency in the A-site cations. This approach has been reported to improve electrochemical performance significantly.^25,26^ Shao's group enhanced both ORR and OER activity by adjusting the A-site cation deficiency in LaFeO_3_ perovskite.^27^ The improvement is attributed to A-site deficiencies that alter the valence state of the B-site transition metal. This modification increases the number of active sites and surface oxygen vacancies, both of which are critical for the ORR.^28–31^ La_0.8_Sr_0.2_MnO_3_ was found to be an effective ORR catalyst with intrinsic activity commensurate with that of commercial Pt/C, particularly when modified with A-site deficiencies.^32–34^

This study details the synthesis of a series of Nd_1−*x*_Sr_*x*_MnO_3_ (*x* = 0.1, 0.2, and 0.3) perovskite oxides *via* a sol–gel process. The resulting 3D structure compounds were evaluated for their electrocatalytic activity for the ORR in an alkaline medium. To characterise their catalytic performance, both rotating disk electrode (RDE) and rotating-ring-disk electrode (RRDE) techniques were used.

The novelty of this study lies in demonstrating how Sr-driven defect in Nd_1−*x*_Sr_*x*_MnO_3_ modifies the concentration of oxygen vacancies and covalency of Mn–O bonds, leading to a combined enhancement in ORR performance and longevity in alkaline medium, emphasizing its promise for use in alkaline fuel cells and metal air batteries. It also reveals a 4e^−^ ORR pathway, with better onset potential with current density nearly equal to that of commercial Pt/C.

### Material synthesis

**Fig. 1:**
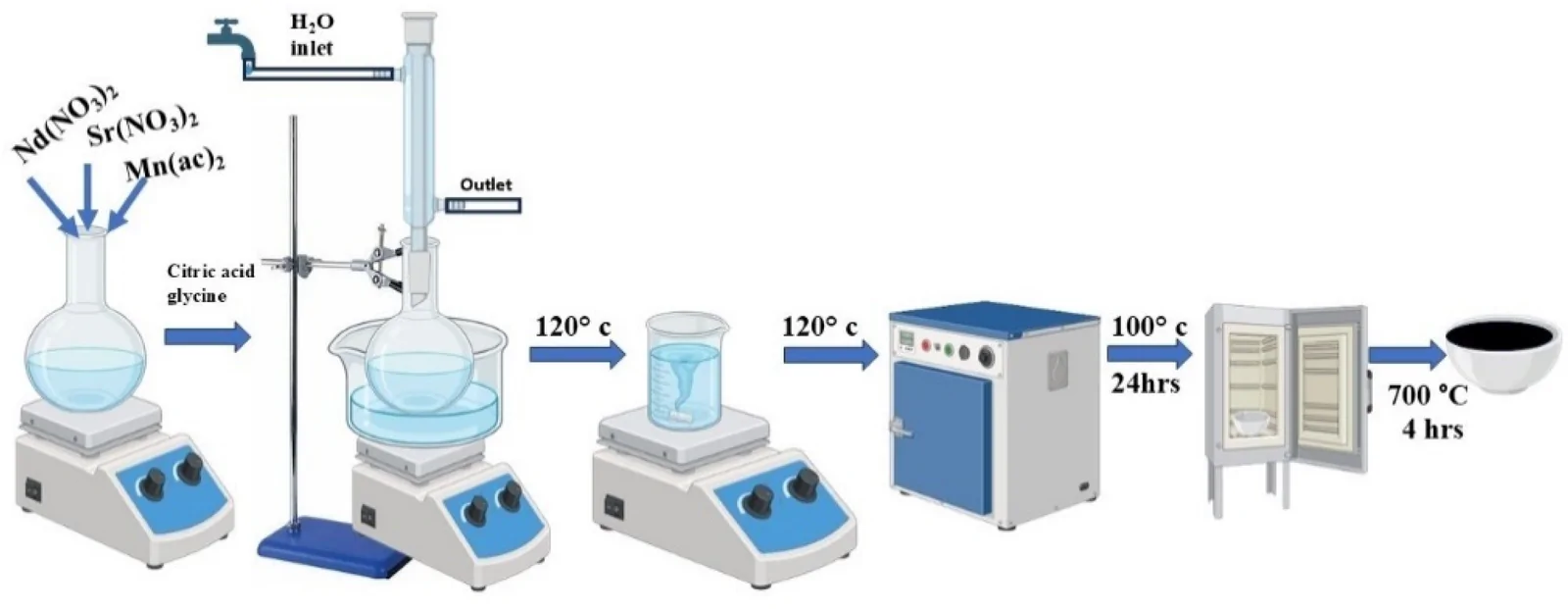
Graphical presentation of material synthesis.

We employed a sol–gel synthesis method to prepare various classes of perovskite oxides with the configuration Nd_1−*x*_Sr_*x*_MnO_3_ (*x* = 0.1, 0.2, 0.3) as shown in Fig. 1. All precursors, including neodymium nitrate (Nd(NO_3_)_2_), strontium nitrate (Sr(NO_3_)_2_), manganese acetate (Mn(ac)_2_), citric acid, and glycine, were purchased from Sigma Aldrich and utilised as received without further purification. The synthesis began by dissolving stoichiometric amounts of the metal salts in separate 10 mL of deionised water. These solutions were then added to a 250 mL round-bottom flask containing 100 mL of water, along with solutions of citric acid and glycine. The molar ratio of citric acid to glycine to total metal ions was maintained at 2 : 2 : 1. The mixture was heated to 120 °C in an oil bath with constant stirring and connected to a condenser. This process continues for 2–3 h. Following this, the solution was then poured into a beaker and gradually heated at 120 °C, where it was held for 24 hours to form a gel. The resulting gel was subsequently dried overnight in an oven at 100 °C, then calcined at 700 °C for 4 hours to yield the final perovskite powders designated as NSM-0.9, NSM-0.8, and NSM-0.7.

### Material characterisation

To determine the crystalline phases of the synthesised perovskites, we used X-ray diffraction (XRD) with a Rigaku Ultima IV instrument. The spectra were obtained using Cu-Kα radiation (*λ* = 1.54178 Å) over a 2*θ* range of 20° to 80° at a scan rate of 4° min^−1^. For morphological characterisation, a Zeiss Gemini SEM 300 field emission scanning electron microscope (FESEM) was used. Particle size was evaluated using an FEI Technai G2S-Twin transmission electron microscope (TEM). Finally, X-ray photoelectron spectroscopy (XPS) with an Al-Kα monochromatic X-ray source was employed to investigate the valence states and surface elemental composition of the materials.

### Electrochemical measurements

We evaluated the electrochemical properties of the catalysts using a Metrohm Autolab 204 RRDE electrochemical workstation that was equipped with a standard three-electrode system. A modified (GC) electrode was used as the working electrode, whereas a graphite rod and an Hg/HgO electrode were used as counter and reference electrode, respectively. The working electrode was prepared by depositing a uniform catalyst ink onto a pre-cleaned GC surface. To formulate the ink, 5 mg of the synthesised NSM perovskite and 1 mg of Vulcan-XC 72 carbon were dispersed in a solution of isopropanol and deionised water in a ratio of 2 : 3. Afterwards, 25 µL of a 0.5% Nafion solution was added and stirred for 30 min. The prepared working electrode had an area of 0.07067 cm^2^ for RDE measurements and 0.196 cm^2^ for RRDE measurements and was dried in a desiccator before use.

We measured both CV and LSV in a 0.1 M KOH solution dripped with either N_2_ or O_2_. These measurements were taken using the RDE at a scan rate of 10 mV s^−1^ with a potential range between 0.067 and 1.067 V *versus* the Reversible Hydrogen Electrode (RHE). We then used RRDE to determine the electron transfer no and the percentage of H_2_O_2_ produced at 1600 rpm with a 10 mV s^−1^ scan rate at 0.5 V. All electrochemical data were ultimately calibrated to the RHE scale utilising the following equation.^35^1
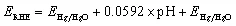
where 
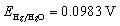
 and pH = 13.

During the evaluation of a catalyst's performance for the oxygen reduction reaction, we paid attention to the following points.

Onset potential (*E*_on_): the onset potential represents the limit value at which the ORR starts to evolve in significant current density values. In this work, the onset potential is defined as the potential at which the current density reaches a value of −0.1 mA cm^−2^.

Electron transfer number (*n*): this tells us the reaction pathway. Ideally, a catalyst should encourage a 4-electron transfer to produce water (H_2_O), not a less efficient 2-electron transfer that creates hydrogen peroxide (H_2_O_2_).

Kinetic current (*J*_K_) and limiting current (*J*_L_): these values measure the speed and maximum rate of the reaction.

Mass activity: these characteristics tell us how much current the catalyst can produce per unit of its mass.

Electrochemical active surface area (ECSA): this measures the total surface area of the catalyst that is actively participating in the reaction.

## Results and discussion

### Structural analysis

**Fig. 2:**
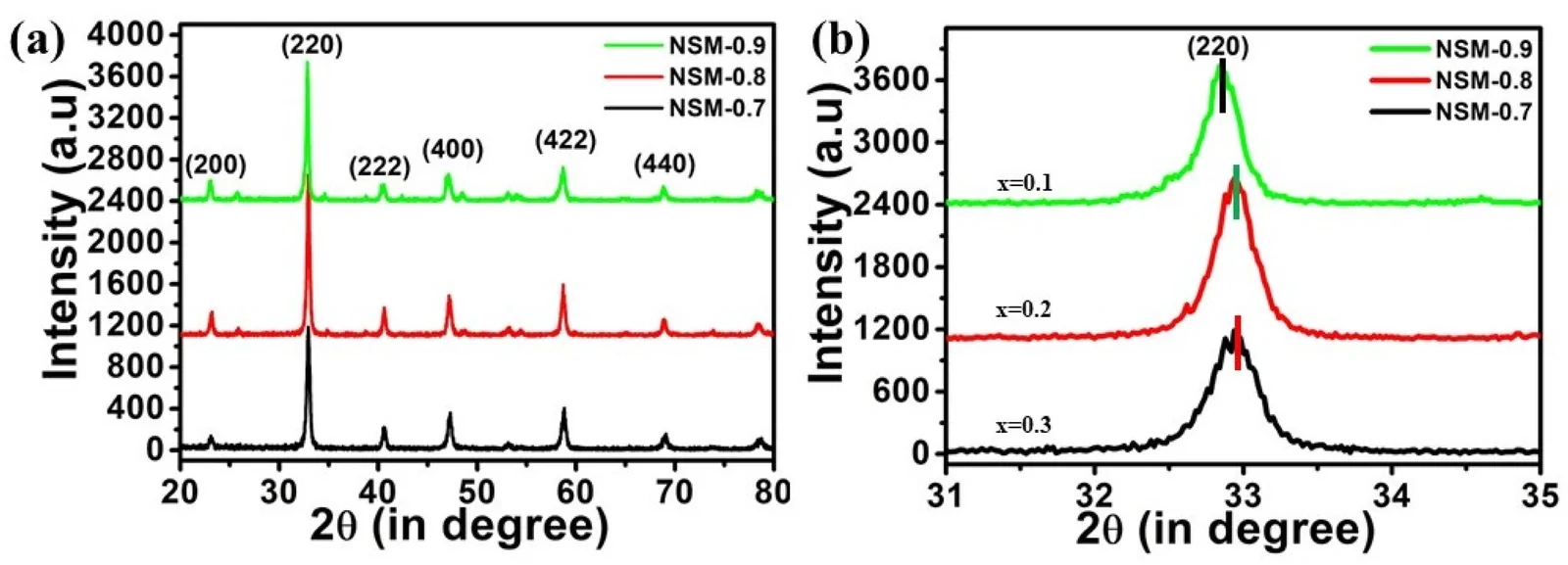
(a) X-ray diffraction spectra of (Nd_1−*x*_Sr_*x*_MnO_3_) (*x* = 0.1, 0.2, 0.3). (b) Magnified XRD spectrum at 2*θ* = 31–35°.

Analysis of the X-ray diffraction (XRD) patterns in Fig. 2a shows that all the synthesised Nd_1−*x*_Sr_*x*_MnO_3_ (NSM) samples, with *x* = 0.1, 0.2, and 0.3, successfully formed the perovskite structure. The sharp and intense reflections indicate high crystallinity, and all peaks are well-indexed to an orthorhombic crystal structure without any detectable impurity phases, suggesting that A-site doping did not significantly alter the fundamental crystal structure.^36^ A detailed view of the highest intensity peaks, centred at 2*θ* = 32.79°, is shown in Fig. 2b. Given the ionic radii of Sr^2+^ (1.26 Å) and Nd^3+^ (1.24 Å) with a coordination number of 8, the substitution of the larger Sr^2+^ ion with the smaller Nd^3+^ ion is expected to result in a contraction of the lattice parameter. Based on Bragg's law, this contraction should cause a shift in the diffraction peak positions towards a higher 2*θ* angle.^37^ However, the introduction of Sr^2+^ cation instead of Nd^3+^ introduces an electronic compensation mechanism, resulting in the partial oxidation of Mn^3+^ to Mn^4+^. The valence fluctuation at the manganese site and the crystallographic disorder introduced at the neodymium site by the strontium substitution combine to cause a complex variation in the lattice parameters. These combined effects are attributed to the observed non-linear or intricate trend in the diffraction peak position.^38^

The particle size of the synthesised perovskites was determined by applying the Debye–Scherrer formula (eqn (2)), which is widely used to estimate crystallite size from X-ray diffraction data. We measure size and *d*-spacing by using the (FWHM) full width at half maximum of the (220) plane, which has maximum intensity among all the peaks. This peak was selected for its high intensity and well-defined shape.2
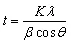


In this formula, the shape coefficient is represented by *K* (0.9), the X-ray wavelength by *λ*, the full width at half-maximum in radians by *β*, and the Bragg angle by *θ*.

The *d*-spacing and crystallite dimensions of the synthesised perovskites were compared in Table 1. The NSM-0.7 sample showed a decrease in both *d*-spacing and crystallite dimensions compared to the other NSM samples.

**Table 1:** Measurement of synthesised particle size and *d*-spacing

Synthesized sample	Particle size (nm)	*d*-spacing (Å)
NSM-0.9	7.29	2.73
NSM-0.8	7.29	2.72
NSM-0.7	6.02	2.72

**Fig. 3:**
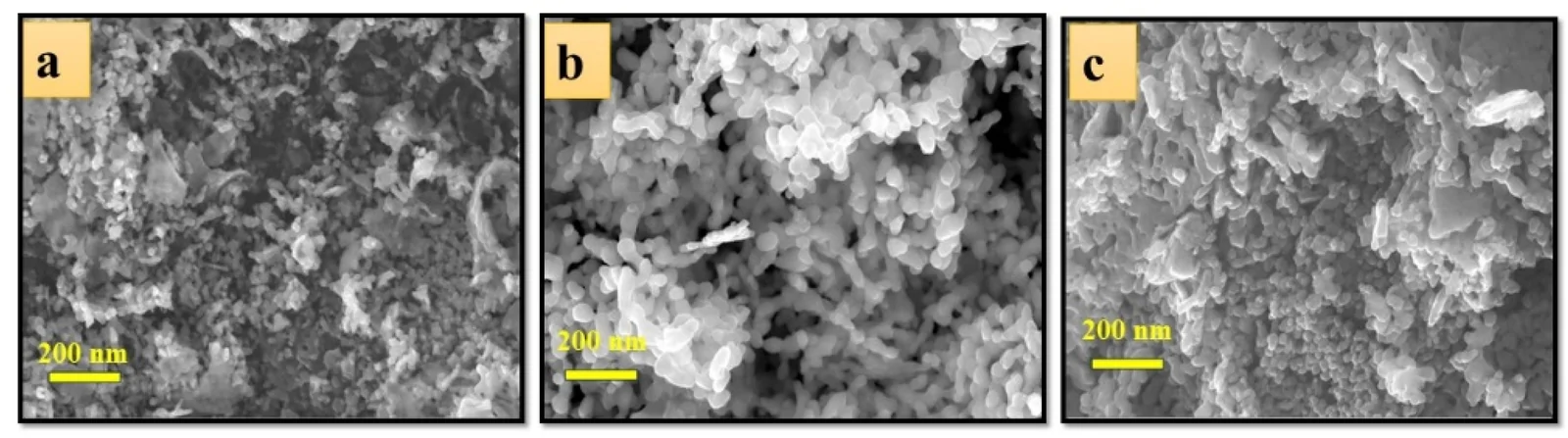
Scanning electron microscope photographs of (a) NSM-0.7 (b) NSM-0.8 (c) NSM-0.9.

As depicted in the Field Emission Scanning Electron Microscope (FE-SEM) images in Fig. 3. All synthesised NSM samples, NSM-0.7, NSM-0.8, and NSM-0.9 in Fig. 3a–c respectively, exhibit a morphology consisting of nanosized particle aggregates. These aggregates, formed during the high-temperature calcination at 700 °C, are a common observation for such materials. Notably, the micrographs reveal that these nanoparticles assemble into a three-dimensional, interconnected network-like structure.

The elemental composition of the Nd_0.7_Sr_0.3_MnO_3_perovskite oxide was examined through EDS spectra. The findings are illustrated in Fig. S1. The detected elements include Nd, Sr, Mn, and O, with no additional elements in the form of impurities present, indicating that a single phase Nd_0.7_Sr_0.3_MnO_3_ was synthesised. Weight percentage and atomic percentage of O K, Mn K, Sr L, and Nd L are 29.1, 22.0, 10.2, 38.7, and also nuclear percentage 69.9, 15.4, 4.5, and 10.3, respectively.

Based on the elements identified (Nd, Sr, Mn, O), the sample is likely a complex oxide material, possibly a perovskite. The EDS analysis confirms the presence of all these constituent elements. The atomic percentages are crucial for determining the stoichiometry of the compound. Also, in Fig. S2 and S4, EDS spectra of NSM-0.8 and NSM-0.9 are given, which confirm that the doping of Sr gradually increases and also the Nd percentage decreases from NSM-0.9 to NSM-0.7.

**Fig. 4:**
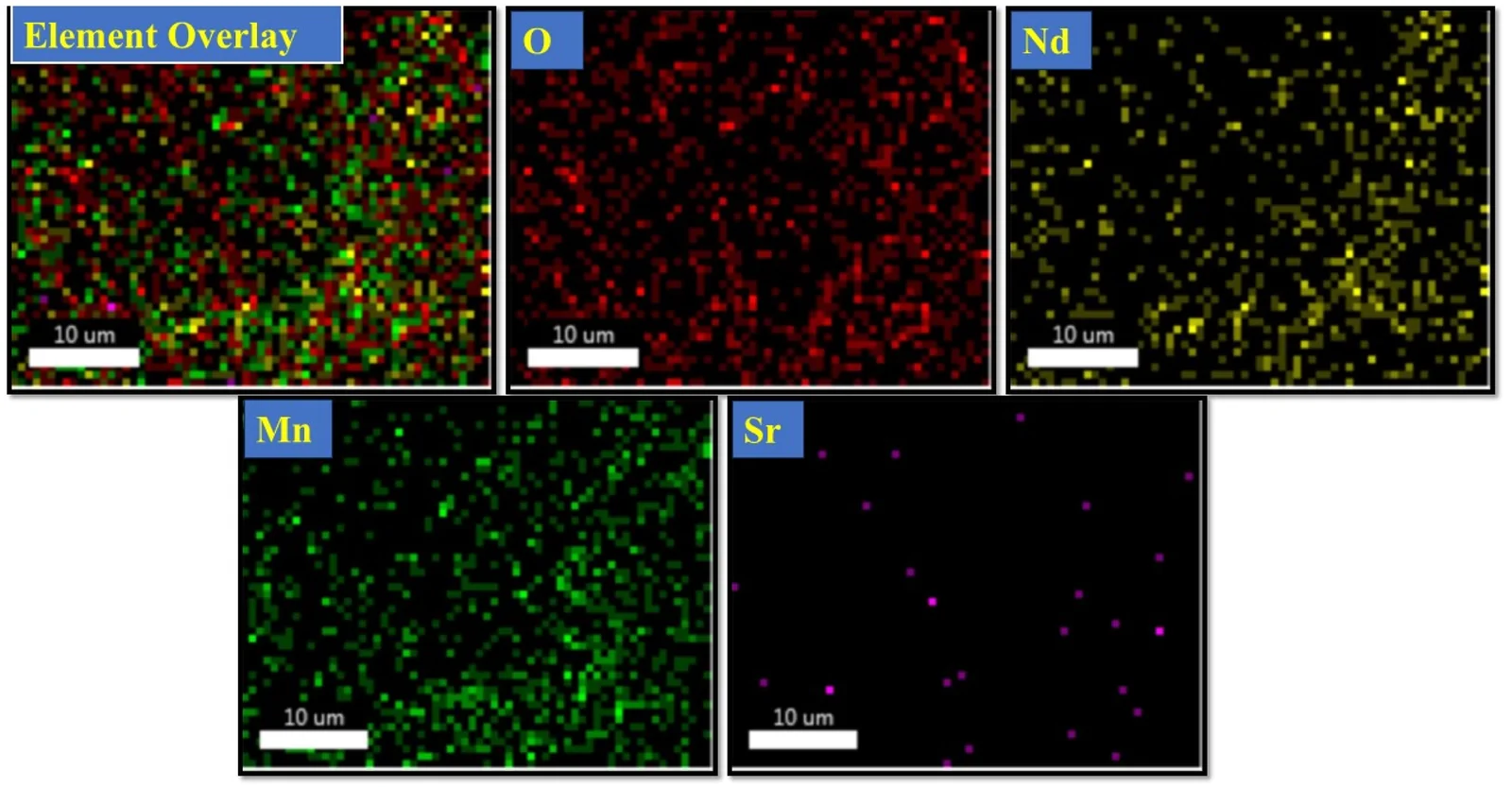
Elemental mapping of NSM-0.7 using EDS.

Fig. 4 illustrates the typical EDS elemental mapping of NSM-0.7. Each element is evenly distributed throughout the perovskite sample. Notably, the even distribution of elements suggests that Sr takes the place of Nd in the A-site. Also, mapping of NSM-0.8 and NSM-0.9 is given in Fig. S3 and S5, which agrees with Sr-doping variation. The elemental mapping reveals that Nd is uniformly spread across the surface of the ceramic, while Sr, Mn, and O accumulate in the grains. As highlighted in previous research,^39^ there are oxygen vacancies located at the grain boundaries (*i.e.*, the darker areas between adjacent grains), which can be seen in the O elemental mapping.

**Fig. 5:**
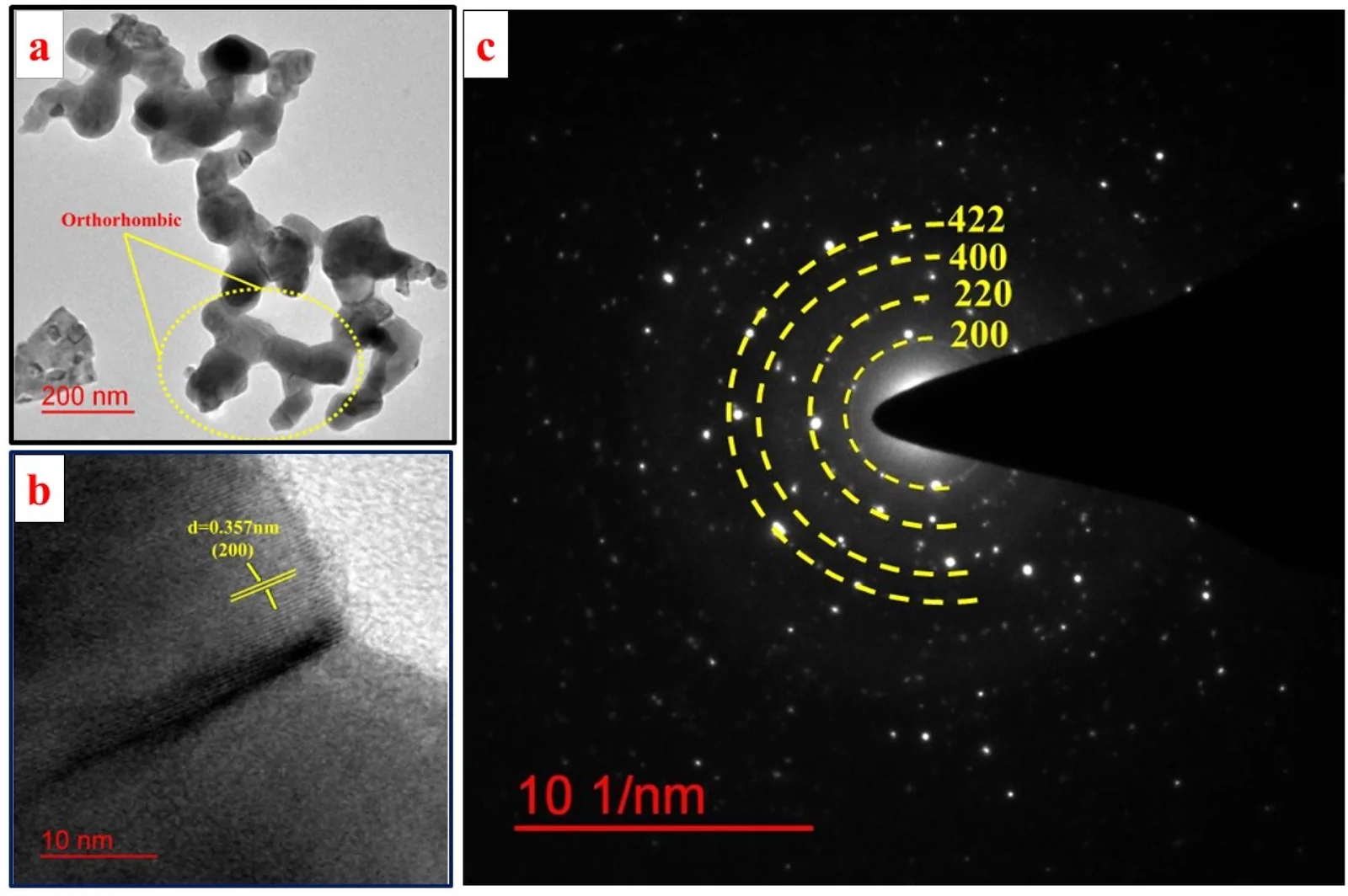
(a) TEM image, (b) HRTEM, (c) SAED of NSM-0.7.

High-resolution transmission electron microscopy (HRTEM) was employed to analyse the structural properties of the materials. Fig. 5a presents a standard TEM image of NSM-0.7, revealing that the nanoparticles are orthorhombic-shaped and agglomerated. The yellow dotted line highlights the orthorhombic crystal system, which is consistent with X-ray diffraction (XRD) data. The HRTEM depicted in Fig. 5b illustrates the grid lines of an individual nanoparticle. The yellow lines indicate an interplanar spacing (*d*-spacing) of 0.357 nm, which is proportional to the (200) crystallographic plane, certifying the material's crystalline nature. Furthermore, the SAED pattern in Fig. 5c consists of concentric rings, characteristic of a polycrystalline material. The dashed yellow circles denote the diffraction rings corresponding to the (200), (220), (400), and (422) planes. The radius of each ring is inversely proportional to the *d*-spacing, which further validates the crystalline structure and phase of the material.

**Fig. 6:**
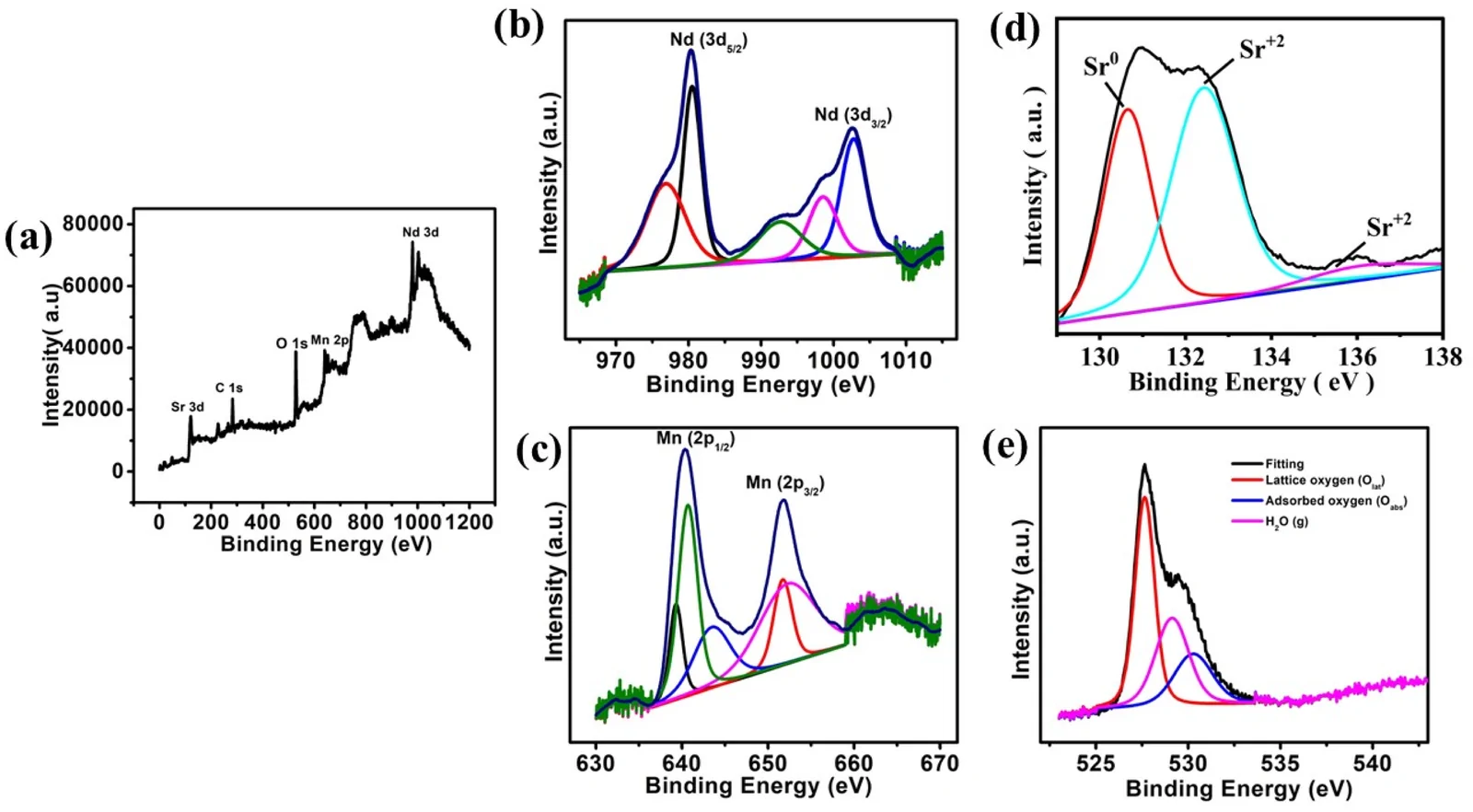
X-ray photoelectron spectroscopy (XPS) of the catalyst Nd_0.7_Sr_0.3_MnO_3_ (a) overall spectrum, and high-resolution curve of (b) Nd 3d region, (c) Mn 2p region and (d) Sm 3d region (e) O 1s region.

X-ray photoelectron spectroscopy (XPS) was performed on the NSM-0.7 sample with (*x* = 0.3) (Fig. 6) to determine the ionic valence states, confirming the exceptional purity of the synthesized perovskite oxide peaks for Nd, Sr, Mn, and O were observed in Fig. 6a, consistent with X-ray diffraction (XRD) and energy dispersive spectroscopy (EDS) results. Specifically, the Nd 3d spectra (Fig. 6b) showed two main peaks at 980.2 eV and 1004.3 eV, which correspond to the Nd 3d_5/2_ and Nd 3d_3/2_ components,^40^ respectively, with a spin–orbit splitting energy difference of approximately 22.1 eV. The NSM-0.7 samples displayed a mixed oxidation state of Neodymium Nd^3+^/Nd^4+^ (Fig. 6b), suggesting that the electrocatalytic oxygen reduction reaction (ORR) performance could be significantly affected by the surface redox interactions of these ions. Furthermore, the manganese (Mn) spectrum (Fig. 6c) features two peaks at 642.3 eV and 653.95 eV corresponding to Mn 2p_3/2_ and Mn 2p_1/2_, respectively,^41^ with a spin–orbit splitting of 11.75 eV, which indicates the presence of a mixed valence state for Mn as well.^42^ This mixed valence is supported by literature references, which associate peaks around 642.05 eV and 653.74 eV with Mn^3+^, and peaks around 644.35 eV and 656.31 eV with Mn^4+^.^43^

The XPS analysis of strontium (Sr) reveals two distinct binding environments in Fig. 6d. The peaks designated as Sr^2+^ appear at higher binding energies, specifically 132.5 eV for Sr 3d_5/2_. This state is attributed to strontium ions incorporated within the crystal matrix or present in stable compounds like strontium oxide SrO or strontium carbonate, where strontium is expected to be in its stable +2 oxidation state.^44^ Conversely, the peaks marked simply as Sr manifest at lower binding energies, centered at 130.5 eV for Sr 3d_5/2_, and are generally assigned to elemental or metallic strontium Sr^0^.

The O 1s XPS spectrum (Fig. 6e) shows three distinct peaks: the peak located at 527.5 eV is assigned to lattice oxygen (O_lat_), while the two higher energy peaks at 529.41 eV and 530.41 eV are attributed to surface-adsorbed oxygen species (O_ads_) and adsorbed water (O_w_), respectively.^45^

The catalytic performance of perovskite materials is heavily influenced by the kind and amount of surface cations, which directly impact the generation of surface oxygen species; a heightened amount of adsorbed oxygen and these characteristic oxygen species can significantly boost electrocatalytic efficiency. These adsorbed oxygen species are often linked to surface hydroxyls^46^ that compensate for missing lattice oxygen, and various studies associate them with oxygen vacancies formed on the surface, which arise because the B-cations are surrounded by only five oxygen anions instead of the typical six.^47^ Surface hydroxyl groups play a critical role in electrochemical reaction mechanisms, and to quantify the beneficial effect of adsorbed oxygen, the ratio of adsorbed to lattice oxygen was calculated, considering only the lattice oxygen at the B-site oxide due to its relevance to electrocatalytic activities. The presence of cobalt can be inferred to promote the formation of adsorbed oxygen species (also known as oxygen vacancies), which subsequently leads to enhanced electrocatalytic performance by increasing the covalency of the M–O bond and boosting the electrical conductivity of the material.^48^

### Electrochemical properties study

**Fig. 7:**
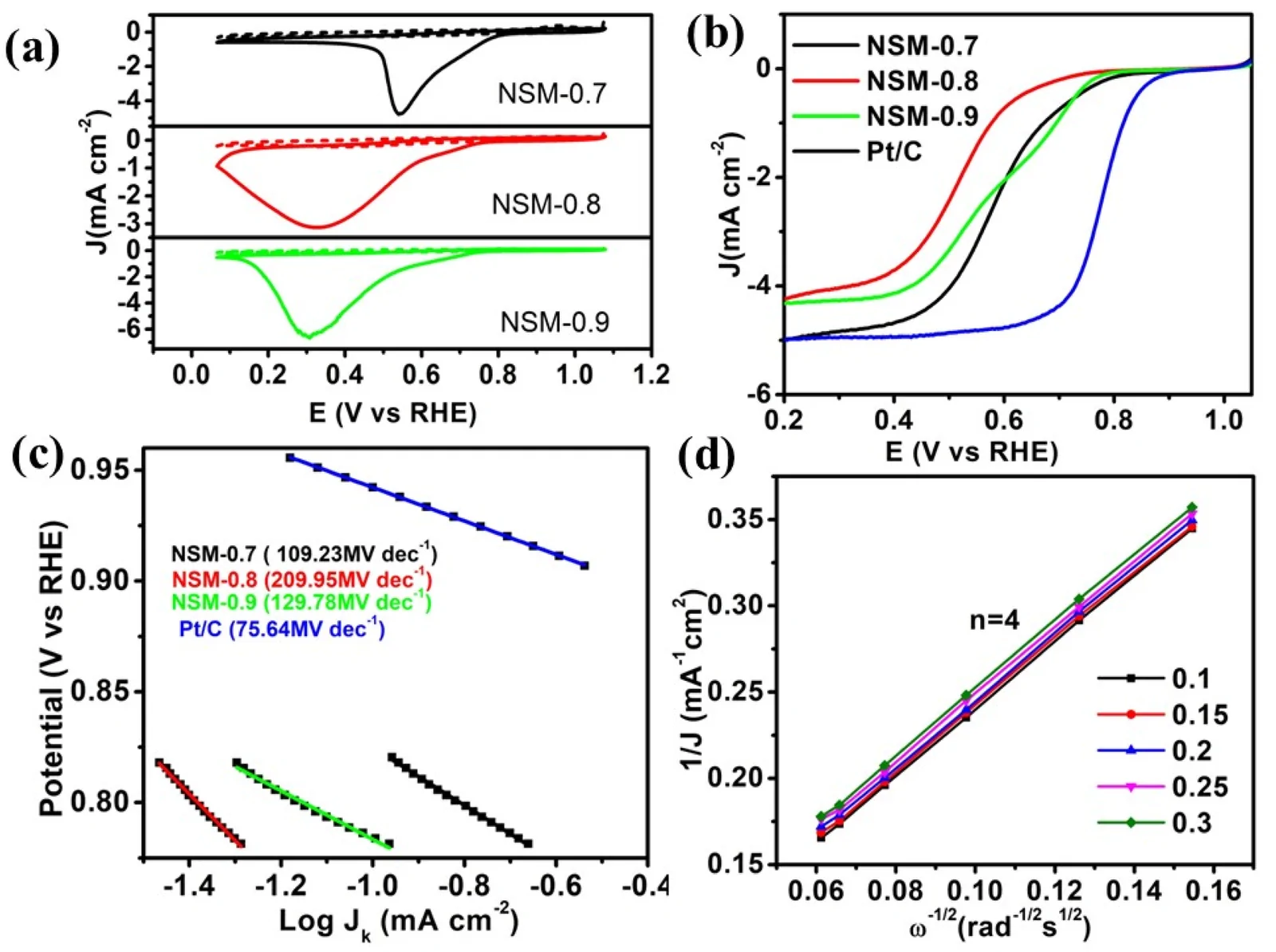
The ORR catalytic performance of different synthesised catalysts, which includes: (a) a comparison of the (CV) graphs for the various catalysts, (b) LSV graphs at a rotation rate of 1600 rpm, (c) Tafel plot for NSM-0.7, and (d) K–L diagrams at multiple electrode potentials for NSM-0.7.

The cyclic voltammetry (CV) curves for the synthesised catalysts (NSM-0.7, NSM-0.8, and NSM-0.9) were measured in a 0.1 M KOH solution saturated with nitrogen. The scan was analysed at a scan rate of 10 mV s^−1^ within the voltage range of 0.076 V to 1.076 V *vs.* RHE. As shown in the plot, no significant reduction peaks were observed, which indicates that the redox processes for these catalysts are negligible. The dotted lines in the plot further highlight this finding. The CV curve for the catalysts, when measured in oxygen-saturated 0.1 M KOH, shows a prominent cathodic peak for oxygen reduction (Fig. 7a, represented by a straight line). The synthesised perovskite demonstrates high efficiency for the ORR. Specifically, the NSM-0.7 catalyst presents the most significant reduction peak at 0.82 V *versus* RHE, indicating that it is the most effective catalyst among the prepared electrocatalysts.

For comparative analysis of the ORR performance between the NSM perovskite catalysts, linear sweep voltammetry (LSV) was performed in a potential range of 0.012 V to 1.012 V at various rotations: 400, 600, 1000, 1600, 2200, and 2500 in 10 mV s^−1^, given in Fig. S6a–c. Then compare the LSV of all NSM catalysts at 1600 rpm with the commercial Pt/C given in Fig. 7b. The onset potential, a key indicator of catalytic activity, was found to vary significantly with increasing strontium (Sr) doping at the A-site of Nd_1−*x*_Sr_*x*_MnO_3_ (NSM). As summarised in Table 2, the onset potential initially increases as the Sr content rises, reaching an optimal value when 30% of the neodymium (Nd) is substituted with Sr. However, any further incorporation of Sr beyond this leads to a decline in the onset potential. This suggests that there is an ideal amount of Sr doping for maximising the catalytic activity of the NSM perovskite. As compared to the benchmark Pt/C catalyst, the NSM catalysts exhibited the following current densities: NSM-0.7: −5 mA cm^−2^, NSM-0.8: −4.23 mA cm^−2^, NSM-0.9: −4.3 mA cm^−2^. Among the synthesised catalysts, NSM-0.7 showed the best performance, with the optimum current density of −5 mA cm^−2^, which is equal to the current density of Pt/C and the highest onset potential, *i.e.*, 0.82 V, which is nearly equal to Pt/C. Additionally, the half-wave potentials (*E*_1/2_), another important metric for catalytic performance, were determined to be: NSM-0.7: 0.58 V, NSM-0.8: 0.51 V, and NSM-0.9: 0.54 V.

**Table 2:** ORR catalytic activity parameters

Catalysts	*E*_on_ (V *vs.* RHE)	*E*_1/2_ (V *vs.* RHE)	*J*_L_ (mA cm^−2^)	*J*_K_ (mA cm^−2^)	Mass activity	*n* (at 0.25V *vs.* RHE)	H_2_O_2_ (%) (at 0.25V *vs.* RHE)	ECSA
NSM-0.7	0.82	0.58	−5	0.41	0.034	3.87	6.81	20.97
NSM-0.8	0.74	0.51	−4.23	0.07	0.005	3.76	12.57	1.66
NSM-0.9	0.78	0.54	−4.3	0.29	0.024	3.74	13.92	9.87

Further investigating the effect of volcanic carbon, we performed LSV at 1600 rpm with different VC amounts in ink and varying loading amounts of 1 mg VC ink, as shown in Fig. S7a and S7b, respectively. We concluded that the VC amount can only influence the current density, with no change in the onset potential. In Fig. S6a, bare VC ink also shows good current. It is confirmed that NSM-0.7 is a better catalyst for ORR. From Fig. S7b, it is confirmed that increasing loading does not affect electrocatalytic activity with respect to surface area.

Additionally, the K–L equation was used to determine Tafel slopes and the quantity of electrons transferred during the ORR, providing valuable insights into the catalytic efficiency of the created NSM-based catalysts.3
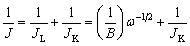

4 *B* = 0.62*nFC*_0_(*D*_0_)^−1/2^*ν*^1/6^

In this equation, *J* represents the observed current density, *J*_L_ indicates the limiting current, and *J*_K_ denotes the kinetic current density. The angular velocity, *ω*, fluctuates between 400 rpm and 2500 rpm, while *D*_0_ stands for the oxygen diffusion coefficient (1.9 × 10^−5^ cm^2^ s^−1^). The variable *n* is the number of electrons transferred per oxygen molecule, *C*_0_ refers to the concentration of oxygen in bulk (1.2 × 10^−3^ mol L^−1^), and *F* symbolizes the Faraday constant (*F* = 96 485 mol^−1^). Additionally, *v* represents the kinematic viscosity of the electrolyte (0.01 m^2^ s^−1^). By analysing the slope of 1/*J* against *ω*^−1/2^, the electron transfer count in the ORR route is determined, which assesses the viability of the reaction.^49^

Tafel plots, which are derived from the Koutecky–Levich (K–L) analysis (eqn (3) and (4)) and depicted in Fig. 7c, were used to assess the kinetic performance of the catalysts. The Tafel slopes were determined to be NSM-0.9: 129.78 mV dec^−1^, NSM-0.8: 209.95 mV dec^−1^, and NSM-0.7: 109.23 mV dec^−1^. From the above synthesized catalysts, NSM-0.7 exhibited the lowest Tafel slope (109.23 mV dec^−1^), as summarized in Table 2. This value is quite close to that of the commercial Pt/C catalyst (75.64 mV dec^−1^), indicating a significantly enhanced ORR rate for the NSM-0.7 catalyst. The improved ORR performance, particularly in NSM-0.7, is directly attributed to the optimal level of strontium (Sr) doping at the A-site of the perovskite structure.

Fig. 7d depicts the K–L diagram for NSM-0.7 at various potentials. This chart illustrates *J*^−1^ about *ω*^−1/2^ for NSM-0.7 under different potentials. The electrons exchanged per O_2_ molecule were assessed by utilizing the slopes of the best linear fit lines. The ORR for the NSM-0.7 sample adheres to first-order kinetics concerning the concentration of dissolved O_2_, as evidenced by the closely aligned and highly linear nature of the graphs. The electron transfer coefficient (*n*) for the NSM-0.7 catalyst is measured as four across varying potentials, as shown by the experimental findings. The kinetic current is calculated from the given eqn (5).^50^5
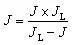
where *J*_lim_ is the limiting current and *J* is the measured current at a given potential of 0.75 V. To reduce the inaccuracies in mass transport adjustments, various catalysts were evaluated based on their kinetic current density within a higher potential range.

The mass-transfer performance of the catalysts was determined by normalizing the kinetic current density (*J*_K_) by the catalyst's mass. The NSM-0.7 catalyst showed the highest kinetic current of 0.41 mA cm^−2^ and a superior mass-transfer performance of 0.034 mA mg^−1^, making it the most efficient among the three samples.

The superior mass-transfer performance and higher kinetic current of NSM-0.7 make it the most effective catalyst among the group. A bar comparison of kinetic current and mass activity is given in Fig. S8a and b.

**Fig. 8:**
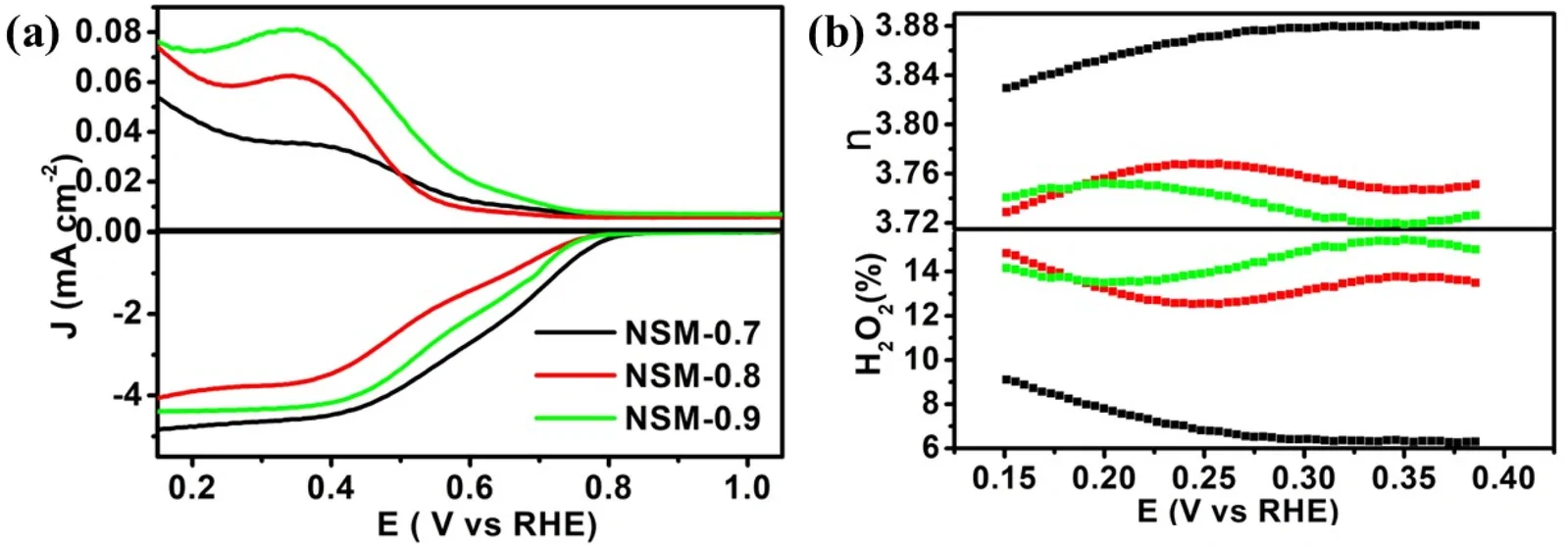
(a) Shows the LSV obtained from RRDE at 1600 rpm, and (b) the synthesised NSM catalyst's HO_2_^−^ (%) production and electron transfer number (*n*) at various potentials.

To evaluate the intrinsic ORR performance, the electrocatalytic behaviour of the synthesized perovskite materials was analysed using a Rotating Ring-Disk Electrode (RRDE), as shown in Fig. 8a. During the RRDE experiment, the platinum (Pt) ring electrode was set at a constant potential of +0.5 V to confirm the oxidation of any hydrogen peroxide (H_2_O_2_) produced. Eqn (6) and (7) were then applied to calculate both the number of electrons transferred per oxygen molecule (*n*) and the percentage of hydroperoxyl radical (H_2_O_2_) generated during the ORR process.6
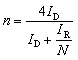
7
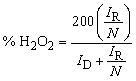


Further, Rotating Ring-Disk Electrode analysis was used to determine the oxygen reduction reaction pathway for a series of NSM perovskite catalysts. By measuring the current at both the glassy carbon (GC) disk electrode (*I*_D_) and the platinum (Pt) ring electrode (*I*_R_), researchers were able to calculate (*n*) and the % (H_2_O_2_) formed. The analysis, summarized in Table 2 and Fig. 8b, revealed that the *n*-values for NSM-0.7, NSM-0.8, and NSM-0.9 were 3.87, 3.76, and 3.74, respectively. Since *n* is approximately equal to 4 and because the percentage of H_2_O_2_ formation was consistently low, the results confirm that all the synthesized NSM catalysts primarily follow the efficient 4-electron pathway. This mechanism is highly beneficial for the ORR, leading to a lower onset potential and a higher current density. Both of these factors are crucial for enhancing catalytic efficiency.

A rapid degradation assessment using cyclic voltammetry demonstrated the impressive long-term stability of NSM-0.7. The catalyst was tested in a 0.1 M KOH solution within the range of 0.067–1.067 V at a scanning speed of 10 mV s^−1^. As shown in Fig. S9a, the cyclic voltammogram of NSM-0.7 showed no significant changes after 500 cycles. Any slight reduction in the limiting current is likely due to minimal Ostwald ripening, a process where smaller carbon nanoparticles co-deposit onto the larger NSM-0.7 nanoparticles.^51^ This slightly reduces the number of available catalytic sites. Further analysis using Field Emission Scanning Electron Microscopy (FESEM) (Fig. S9b) confirmed that the NSM-0.7 particles showed minimal agglomeration after 500 ORR test cycles compared to the initial sample. X-ray Diffraction (XRD) analysis (Fig. S9c) revealed that after 500 cycles, the catalyst's crystallinity had decreased, but its phase remained unchanged. An additional hump observed between 2*θ* = 20° and 30° is attributed to the presence of Vulcan carbon, which was mixed with the NSM material during catalyst preparation. This combined evidence confirms the excellent long-term stability of the NSM-0.7 nanoparticles.

**Fig. 9:**
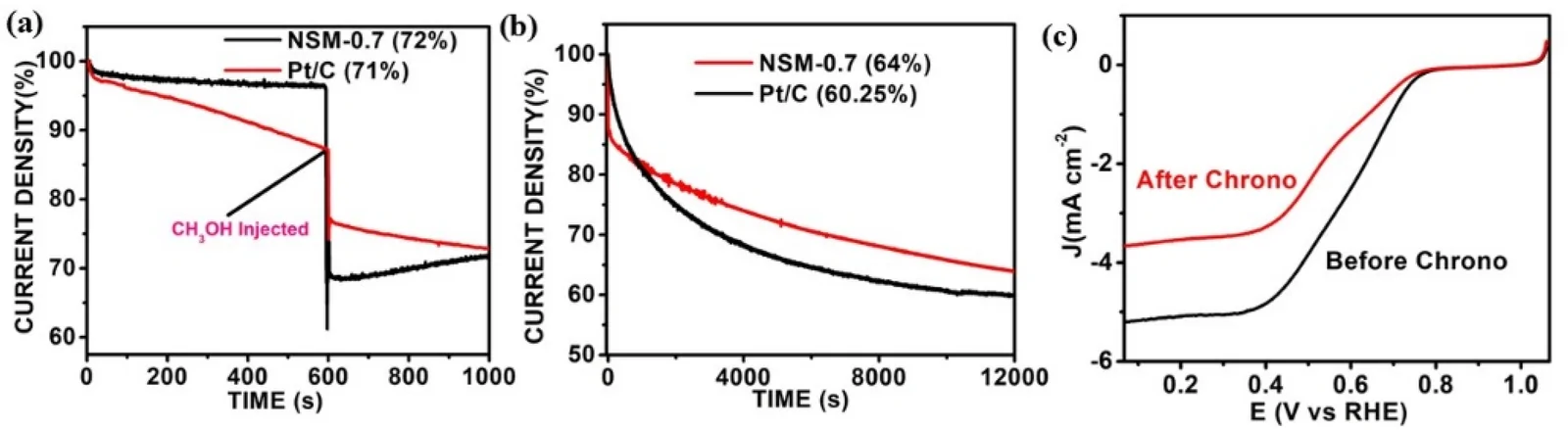
(a) NSM-0.7's tolerance to methanol and (b) chronoamperometry response at 1600 rpm for the revolving disk electrode in 0.1 M KOH. (c) A comparison of LSV before and after chronoamperometric analysis.

The stability and methanol crossover tolerance of any electrocatalyst are crucial for its practical application. Fig. 9a shows the chronoamperometric behaviour of the NSM-0.7 catalyst. After methanol was introduced, the catalyst's performance recovered in about 600 seconds, showing it was unaffected by the methanol crossover effect. In contrast, the commercial Pt/C catalyst showed a significant shift in current from negative to positive after methanol injection, indicating the start of the Methanol Oxidation Reaction (MOR). These results verify that the synthesized NSM-0.7 electrocatalyst has a superior tolerance to methanol crossover, with a capacity of 72%.^52,53^ Further durability tests, shown in Fig. 9b, revealed that the NSM-0.7 catalyst degrades much more slowly than the commercial Pt/C catalyst. After 12 000 seconds, NSM-0.7 maintained an impressive 64% of its initial current, whereas the ORR current on the Pt/C catalyst declined sharply during the same duration under equivalent conditions. To further confirm its stability, the Linear Sweep Voltammetry (LSV) results before and after the chronoamperometry test were compared in Fig. 9c. The minimal change in both the onset potential and the limiting current further validates the stability of the NSM-0.7 catalyst. These results collectively confirm that the synthesized NSM-0.7 electrocatalyst is both stable and highly resistant to methanol crossover, making it a promising candidate to be used in direct methanol and alkaline fuel cells.

**Fig. 10:**
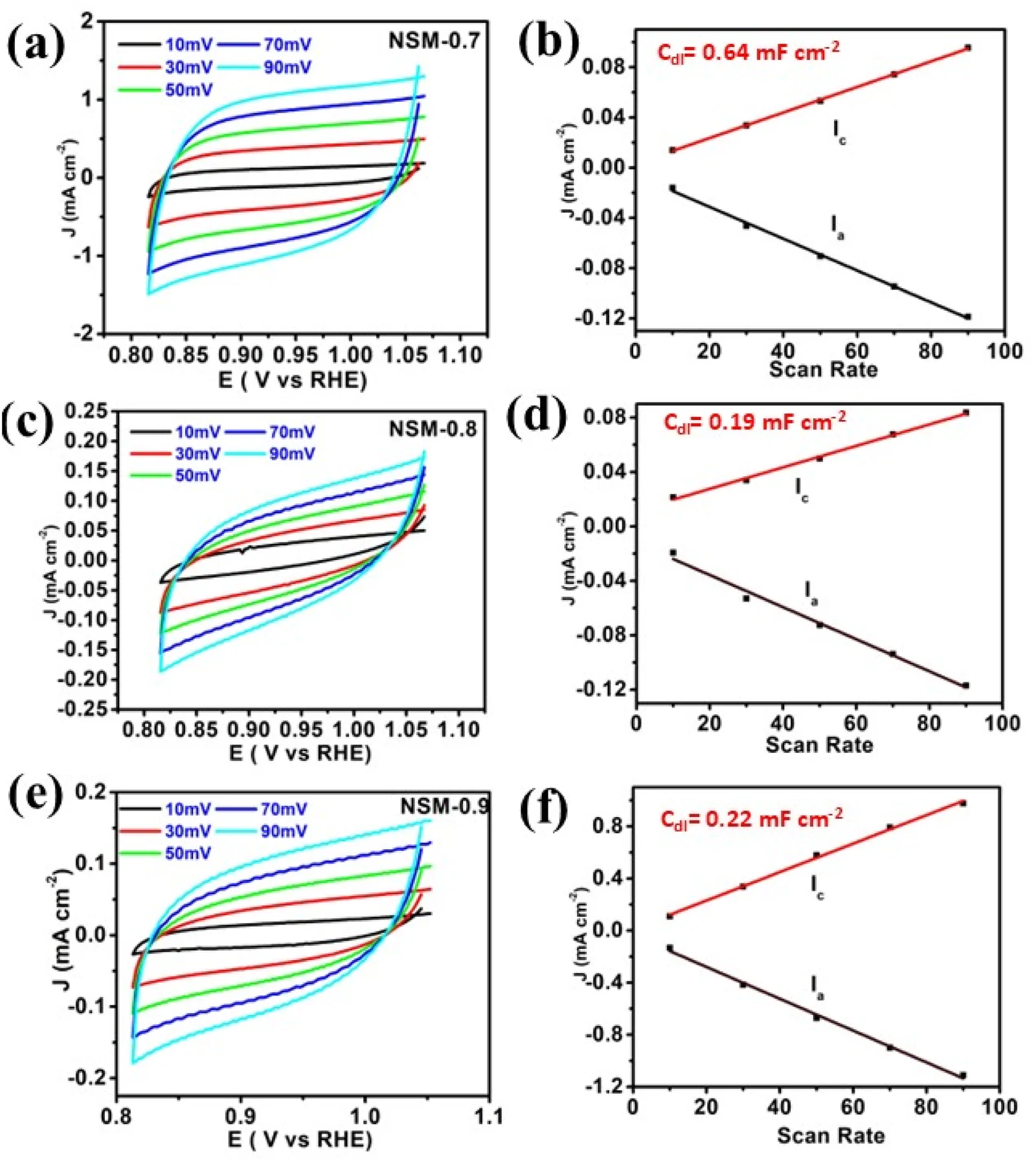
A linear plot of capacitive current density against scan rate (at 0.9 V *vs.* RHE) for (b) NSM-0.7, (d) NSM-0.8, and (f) NSM-0.9 electro-catalysts is shown in, CV curves of (a) NSM-0.7, (c) NSM-0.8, and (e) NSM-0.9 electro-catalysts recorded at different scan speeds (10–90 mV s^−1^) within a potential range of 0.815–1.067 V RHE.

We assessed the electrochemically responsive surface area (ECSA) of the synthesized materials by analysing the double-layer capacitance (*C*_dl_) of the catalytic framework. Cyclic voltammetry (CV) curves were acquired in the non-faradaic region for the NSM-0.7, NSM-0.8, and NSM-0.9 catalysts Fig. 10a, c and e, respectively. The measurements were performed at various scan rates ranging from 10 to 90 mV s^−1^ within a voltage window of 0.82–1.17 V. In this voltage range, the only source of transient non-faradaic current is the charging and discharging of the double electric layer at the electrode–electrolyte interface.^54^ As shown in Fig. 10, the charge and discharge currents increase linearly with increasing scan rates. The *C*_dl_ values were derived from the linear plots of capacitive current density *versus* scan rate, specifically at 1 V *vs.* RHE (Fig. 10b, d and f). By analysing the non-faradaic current, which is associated with the adsorption and desorption behaviour on the electrode surface, we can determine the actual surface area of the electrode.^55,56^

To calculate the ECSA for the working electrode, its roughness factor (*R*_f_) needs to be computed using eqn (8).

8 ECSA = *R*_f_·*S*

where “*S*” was the surface area of the electrode (*S* = 0.07067 cm^−2^). By using eqn (9), *R*_f_ was calculated from *C*_dl_.

9 *R*_f_ = *C*_dl_/0.040 cm^−2^

The double-layer capacitance (*C*_dl_) values calculated for the catalysts were found to be 0.64 mF cm^−2^ (NSM-0.7), 0.19 mF cm^−2^ (NSM-0.8), and 0.22 mF cm^−2^ (NSM-0.9). These values correspond to electrochemically active surface areas (ECSA) of 113.07, 34.48, and 39.75 cm^2^, respectively. Contrary to the expected trend where a higher ECSA corresponds to a more active catalyst, NSM-0.7, having the highest *C*_dl_ value, demonstrates the highest catalytic efficiency. This suggests that a larger surface area is generally beneficial and may be more significant in determining the overall performance of the NSM-0.7 electrocatalyst.^57^ Electrochemical Impedance Spectroscopy (EIS) is a powerful method used to study charge transfer dynamics and diffusion processes at the electrode–electrolyte interface. The Nyquist plots in Fig. S10 illustrate the results for the synthesized electrocatalysts. The inset figure displays an equivalent circuit schematic used to analyse the experimental data. This circuit consists of: *R*_s_: resistance of the solution, *W*: Warburg impedance, which demonstrates diffusion, *R*_ct_: charge-transfer resistance, and CPE: constant phase element, which accounts for non-ideal capacitance.^58^ The *R*_ct_ values for NSM-0.7, NSM-0.8, and NSM-0.9 were determined to be 53.13 Ω, 54.42 Ω, and 53.13 Ω, respectively. Finally, we compare a series of Sr-doped perovskites' ORR catalytic activity based on factor *E*_on_, *E*_1/2_, and *J*_L_ given in Table 3.

**Table 3:** Comparison of ORR activity of perovskites

Catalysts	*J*_L_ (mA cm^−2^)	*E*_onset_ (V *vs.* RHE)	*E*_1/2_ (V *vs.* RHE)	References
**NSM-0.7**	**−5**	**0.82**	**0.58**	**This work**
**NSM-0.8**	**−4.23**	**0.74**	**0.51**	
**NSM-0.9**	**−4.3**	**0.78**	**0.54**	
(La_0.7_Sr_0.3_)_0.98_MnO_3_	5.3	0.84	—	30
LSM-CeO_2_	5.7	0.881	0.666	31
LSM	5.5	0.857	0.642	
(La_0.8_Sr_0.2_)_0.95_Mn_0.5_Fe_0.5_O_3_	4.5	0.824	—	32
HS-La_0.8_Sr_0.2_MnO_3_	6.46	0.827	0.638^c^	33
C-La_0.8_Sr_0.2_MnO_3_	4.94	0.765	0.602^c^	
U-La_0.8_Sr_0.2_MnO_3_	5.14	0.78	0.609^c^	
Co-OEP/LSMF/C	4	0.906	0.78	59
La_0.8_Sr_0.2_Mn_0.6_Ni_0.4_O_3_	4.43	0.868	0.628^b^	60
La_0.8_Sr_0.2_MnO_3_	3.28	0.838	0.618^b^	
La_0.8_Sr_0.2_Mn_0.8_Ni_0.2_O_3_	3.63	0.848	0.628^b^	
La_0.8_Sr_0.2_MnO_3_ nanorod	6.3	0.834	0.66^c^	61
La_0.8_Sr_0.2_MnO_3_ particle	2.93	0.7	0.638^c^	
LaMnO_3_ film	—	0.825	—	62
La_0.67_Sr_0.33_MnO_3_ film	—	0.825	—	
La_0.4_Sr_0.6_MnO_3_	1.77	0.765	0.465^a^	63
La_0.2_Sr_0.8_MnO_3_	1.37	0.665	0.395^a^	
La_0.8_Sr_0.2_MnO_3_	1.47	0.565	0.415^a^	
La_0.6_Sr_0.4_MnO_3_	1.52	0.565	0.415^a^	
La_0.8_Sr_0.2_MnO_3_	4.5	0.746	0.606^c^	64
La_0.8_Sr_0.2_MnO_3−*d*_ film	—	0.73	—	65
30%Pd-decorated La_0.6_Sr_0.4_CoO_3−*d*_	1.5	0.945	—	66

a Converted from Hg/HgO electrode (*E vs.* RHE, *E vs.* MMO + 0.098 V + 0.059 × pH).

b Converted from saturated calomel electrode (*E vs.* RHE, *E vs.* SCE + 0.241 V + 0.059 × pH).

c Converted from Ag/AgCl in 3 M KCl (*E vs.* RHE, *E vs.* Ag/AgCl + 0.209 V + 0.059 × pH).

## Conclusion

A series of Nd_1−*x*_Sr_*x*_MnO_3_ electrocatalysts with *x* = 0.1, 0.2, and 0.3 was synthesized *via* a sol–gel technique and characterized using various methods, including X-ray Diffraction (XRD), Scanning Electron Microscopy (SEM), Transmission Electron Microscopy (TEM), and X-ray Photoelectron Spectroscopy (XPS). The resulting NSM-0.7 catalyst specifically showed an orthorhombic structure with a particle size of 6.02 nm and a *d*-spacing of 2.72 nm. Furthermore, Field Emission Scanning Electron Microscopy (FESEM) analysis confirmed that this catalyst possessed a distinctive dual-triple-layer, three-dimensional web-like structure. TEM analysis indicated particles ranging from 5 to 20 nm. NSM-0.7 emerged as the most proficient ORR electrocatalyst among all the synthesized materials, demonstrating an onset potential of 0.082 *vs.* Hg/HgO, a kinetic current *J*_K_ of 0.41 mA cm^−2^ at 0.73 V, and a Tafel slope of 109.23 mV dec^−2^, accompanied by a limiting current *J*_L_ of −5 mA cm^−2^. The catalytic stability of NSM-0.7 is credited to its capacity to host cations in the A positions. According to chronoamperometry results, the NSM-0.7 electrocatalyst showcases impressive durability and outstanding methanol resistance, comparable to the commercial Pt/C. NSM-0.7 was recognized as the most powerful catalyst among the synthesized perovskite materials.

## Conflicts of interest

There are no conflicts to declare.

## Supplementary Material

RA-016-D6RA00332J-s001

## Data Availability

The data supporting this article have been included as part of the supplementary information (SI). Supplementary information is available. See DOI: https://doi.org/10.1039/d6ra00332j.
