# Supplementary material for: Maximal electrocatalytic activity of Sr-doped Nd1−xSrxMnO3 perovskite oxides (x = 0.1, 0.2, 0.3) towards ORR: for next-generation power systems
Source: RSC Adv. 2026 Mar 10;16(15):13408–19. doi: 10.1039/d6ra00332j (PMC12974575; doi:10.1039/d6ra00332j)
Supplement: RA-016-D6RA00332J-s001 [file RA-016-D6RA00332J-s001.pdf]

Supplementary Information (SI) for RSC Advances.
This journal is © The Royal Society of Chemistry 2026

Supporting information

Maximal Electrocatalytic Activity by Sr-doped, $Nd_{1-x}Sr_xMnO_3$ Perovskite Oxides (x = 0.1, 0.2, 0.3) towards ORR: for Next-Generation Power Systems

B.B. Nayak[a], Rakesh Ranjan Das[a], P.Parida[a], P. Parhi*[a]

[a] Department of Chemistry, Ravenshaw University, Cuttack, Odisha-753003, India.

*E-mail[a]: pparhi@ravenshawuniversity.ac.in, Phone: +91-8895193144

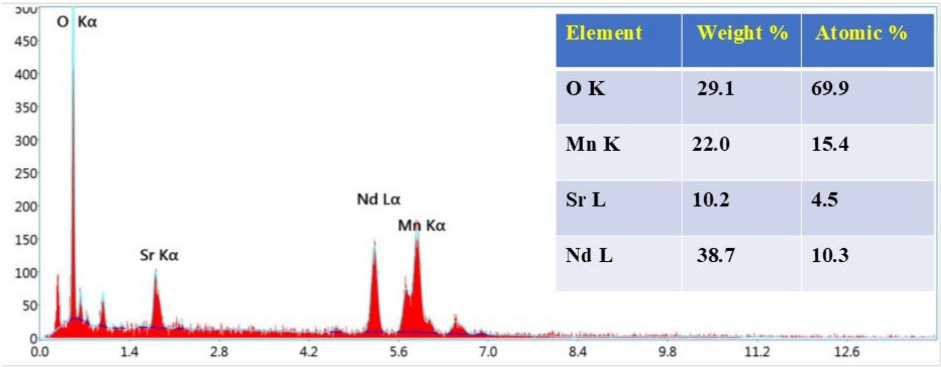

Element	Weight %	Atomic %
O K	29.1	69.9
Mn K	22.0	15.4
Sr L	10.2	4.5
Nd L	38.7	10.3

Figure S1 EDS spectra of the NSM-0.7

The elemental composition of the $Nd_{0.7}Sr_{0.3}MnO_3$ perovskite oxide was examined through EDS spectra. The findings are illustrated in Figure S1. The detected elements include Nd, Sr, Mn, and O, with no additional elements in the form of impurities present, indicating that a high-quality $Nd_{0.7}Sr_{0.3}MnO_3$ was synthesized. Weight percentage and atomic percentage of O K, Mn K, Sr L, and Nd L are 29.1, 22.0, 10.2, 38.7, and also nuclear percentage 69.9, 15.4, 4.5, and 10.3, respectively.

Based on the elements identified (Nd, Sr, Mn, O), the sample is likely a complex oxide material, possibly a perovskite. The EDS analysis confirms the presence of all these constituent elements. The atomic percentages are crucial for determining the stoichiometry of the compound.

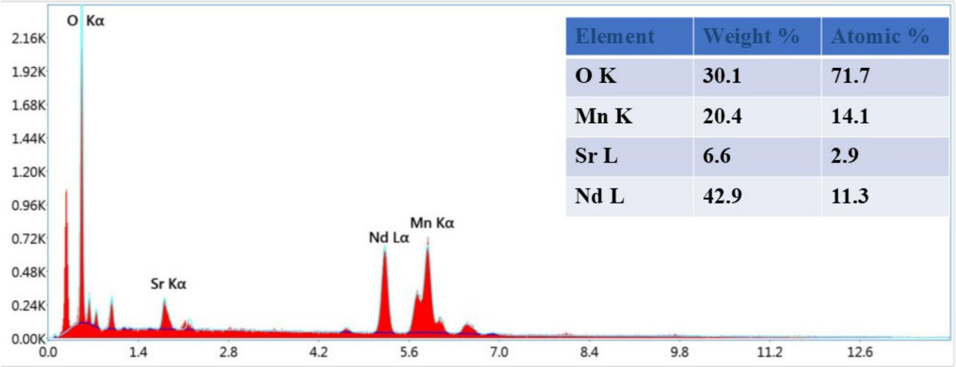

Figure S2. EDS elemental distribution content of NSM-0.8

Figure S2. displays the results of an Energy-Dispersive X-ray Spectroscopy (EDS) analysis, a technique used to determine the elemental composition of NSM-0.8. The EDS spectrum displays distinct peaks corresponding to the characteristic X-rays emitted by the elements present, namely Oxygen (O K), Strontium (Sr K), Neodymium (Nd L), and Manganese (Mn K). The insert table quantifies the elemental composition, indicating that the material is primarily composed of O K, Mn K, Sr L, and Nd L, are 30.1, 20.4, 6.6, and 42.9, and also atomic percentages of 71.7, 14.1, 2.9, and 11.3, respectively.

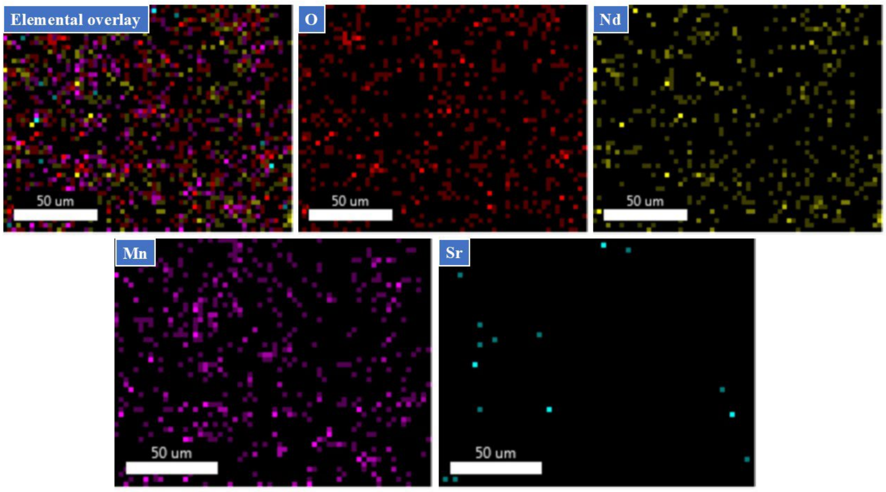

Figure S3. EDS elemental distribution mapping of NSM-0.8

Figure S3 displays the results of an Energy-Dispersive X-ray Spectroscopy (EDS) mapping analysis of NSM-0.8, which visually illustrates the spatial distribution of different elements within a sample at the microscopic level. Figure S3 displays the results of an Energy-Dispersive X-ray Spectroscopy (EDS) mapping analysis of NSM-0.8, which visually illustrates the spatial distribution of different elements within a sample.

The individual maps confirm the relative homogeneity of the major components: Oxygen (O) is widely and uniformly distributed (red), as is Neodymium (Nd) (yellow), indicating they are primary constituents of the material's bulk structure. Manganese (Mn) (magenta) also shows a relatively dense and uniform distribution, suggesting it is well-incorporated into the material. In contrast, Strontium (Sr) (cyan) is present in a much more sparse and localized manner, appearing as only a

few distinct spots, which suggests either a very low overall concentration or that it exists as isolated minority phases or segregates on the sample's surface.

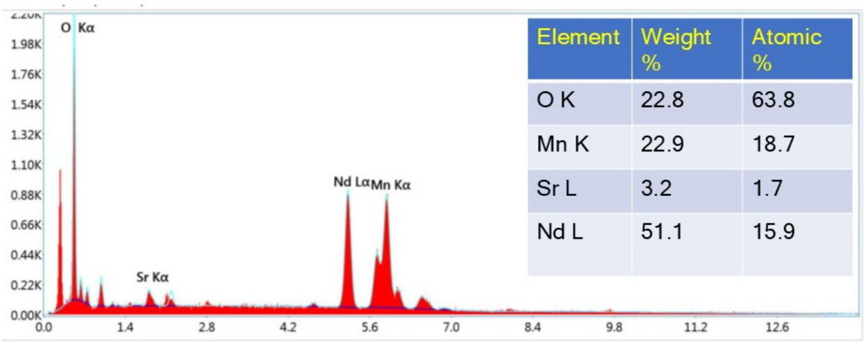

Element	Weight %	Atomic %
O K	22.8	63.8
Mn K	22.9	18.7
Sr L	3.2	1.7
Nd L	51.1	15.9

Figure S4. EDS elemental distribution content of NSM-0.9

Figure S4. The provided shows a graph with several labeled peaks and a table below it. This is a typical output from an Energy-Dispersive X-ray Spectroscopy (EDS) analysis, a technique used to determine the elemental composition of a sample. Each sharp peak corresponds to the characteristic X-rays emitted by a specific element when it is bombarded with a high-energy electron beam, and the energy of these X-rays is unique to each element. The peaks are labeled to identify the elements present. In this figure, there are prominent peaks for carbon (C) and oxygen (O). Smaller peaks are visible for manganese (Mn) and dysprosium (Dy). The labels also indicate the specific electron shell transition (e.g., Kα, Lα) that produced the X-ray. For example, the Mn Kα peak results from a transition to the K-shell of a manganese atom.

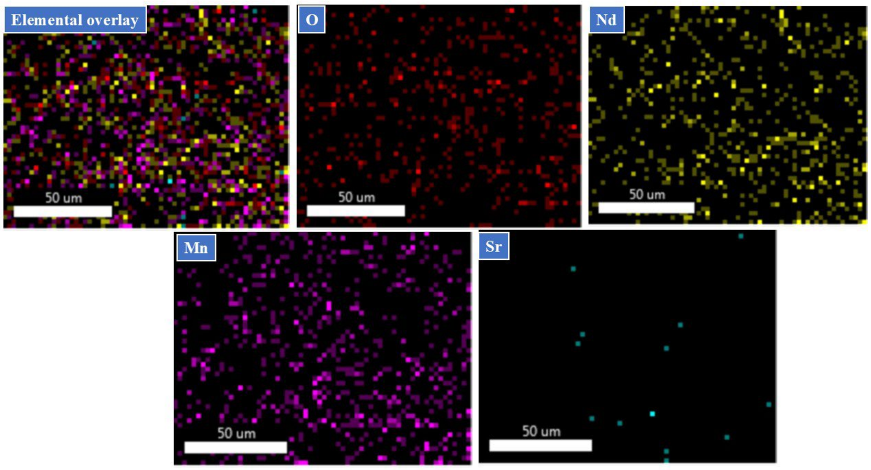

Figure S5. EDS elemental distribution mapping of NSM-0.9

Figure S5. shows an elemental mapping analysis of a material, likely performed using Energy-Dispersive X-ray Spectroscopy (EDS), which is a common technique used with scanning electron microscopes. Oxygen, Neodymium, and Manganese appear to be homogeneously distributed across the entire mapped area, indicating that the bulk material is composed of a complex oxide containing these three elements. In contrast, Strontium shows a much lower density of signal points, suggesting it is either a minor dopant or is concentrated in a few discrete locations rather than uniformly distributed throughout the matrix.

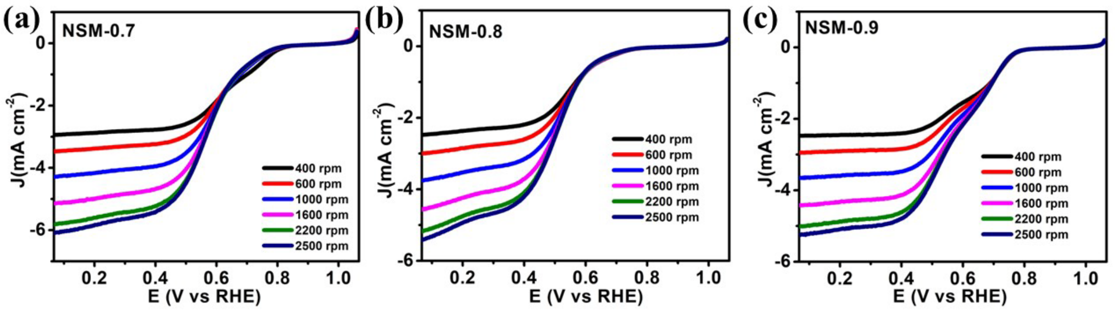

Figure S6. Linear sweep voltammograms curves of (a) NSM-0.7, (b) NSM-0.8, (c) NSM-0.9 at scan rates of 10 mV/s and rotation speeds of 400, 600, 1000, 1600, 2200, and 2500 rpm.

This experiment showed that the prepared catalysts displayed sensitive responses for the electrochemical behaviour towards O_2 reduction. The linear sweep voltammograms (LSV) curves at various rotation rates, i.e., 400, 600, 1000, 1600, 2200, and 2500, of the prepared catalysts in an O_2-saturated 0.1 M KOH solution at a scan rate of 10 mV s^{-1} are shown in Figure S6 (b, d, f, and h). All of the synthesized catalysts displayed stable diffusion plateau areas, as shown in Figure S6, and the current density increased steadily as the rotational speed increased.

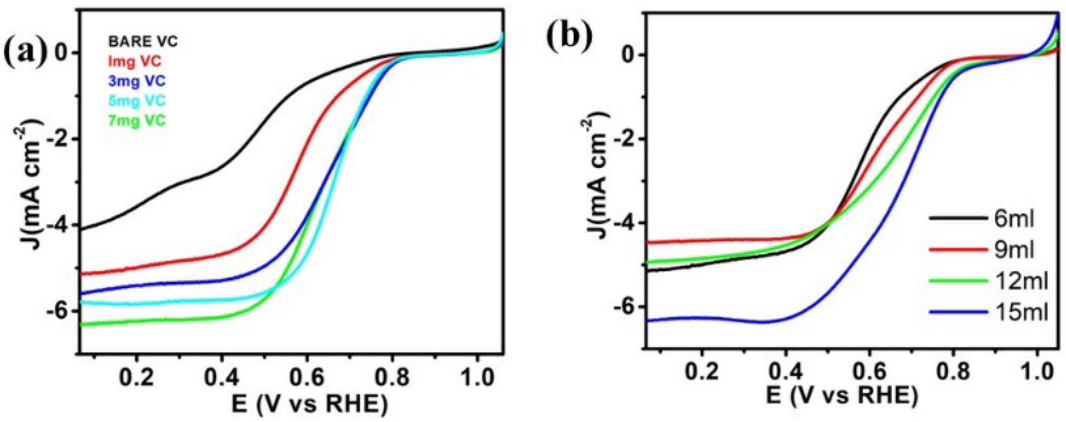

Figure S7. LSV comparison by taking various amounts of volcano carbon (a) LSV comparison of different amounts of loading of 1mg VC ink.

This is Figure S7 (a) presents Linear Sweep Voltammetry (LSV) curves from the study of Oxygen Reduction Reaction (ORR), investigates the effect of the loading of a material based on VC (different VC-supported catalyst) on the catalytic performance, showing that increasing the VC loading from up to shifts the onset potential is constant but the current density increases, indicating enhanced electrocatalytic activity compared to the bare VC. Figure S7 (b) shows the effect of varying a volume parameter (indicated by) on the same reaction, where appears to offer the best performance with the most positive half-wave potential and a high limiting current density, suggesting that both catalyst loading and the volume parameter are critical factors influencing the overall efficiency and kinetics of the electrochemical reaction.

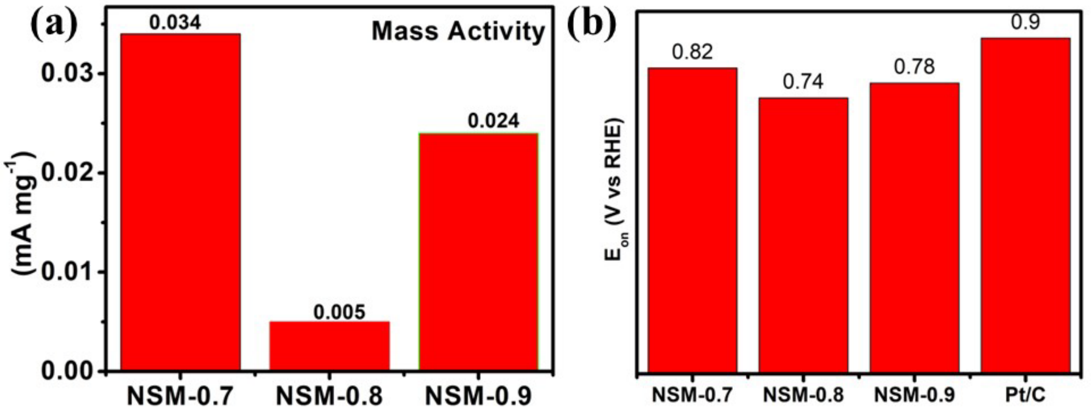

Figure S8. (a) A bar graph comparing the onset potential and (b) Comparison of kinetic current density of the prepared electrocatalysts.

In Figure S8, the electrocatalytic performance of three different materials, i.e, NSM-0.7, NSM-0.8, and NSM-0.9 with Pt/C, for the Oxygen Reduction Reaction (ORR). Figure S8 (a) presents the Mass Activity (mA mg^{-1}), showing that NSM-0.7 exhibits the highest mass activity, 0.034 mA mg-1, significantly NSM-0.8 (0.005 mA mg^{-1}) and NSM-0.9 (0.024 mA mg^{-1}), indicating it's the most efficient catalyst based on catalyst mass. Figure S8 (b) compares the Onset Potential E_{on}, where Pt/C has the highest onset potential 0.9 V, followed by NSM-0.7, 0.82 V, and NSM-0.9, 0.78 V, suggesting that while Pt/C initiates the reaction at the most favourable potential, NSM-0.7 is the best performing non-Pt catalyst in terms of both mass activity and having an onset potential relatively close to the benchmark.

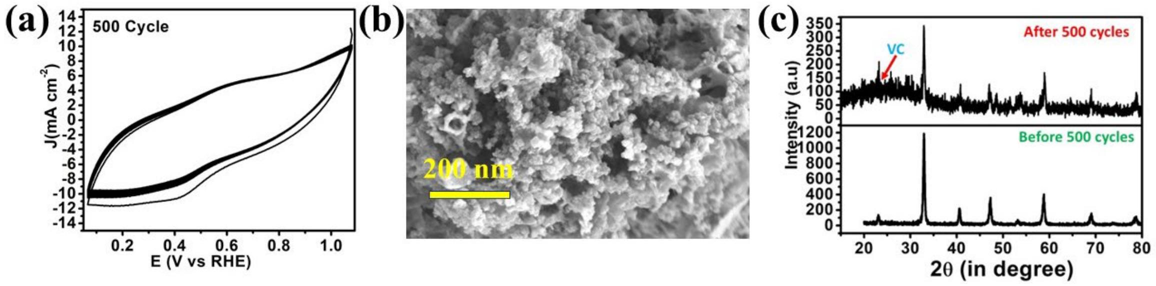

Figure S9. (a) CV, (b) FESEM, and (c) PXRD graphs after 500 cycles of ORR at 10 mV/S scan rate.

A rapid degradation assessment using cyclic voltammetry demonstrated the impressive long-term stability of NSM-0.7. The catalyst was tested in a 0.1 M KOH solution within the range of 0.067-1.067 V at a scanning speed of 10 mV/s. As shown in Figure S9a, the cyclic voltammogram of NSM-0.7 showed no significant changes after 500 cycles. Any slight reduction in the limiting current is likely due to minimal Ostwald ripening, a process where smaller carbon nanoparticles co-deposit onto the larger NSM-0.7 nanoparticles. This slightly reduces the number of available catalytic sites. Further analysis using Field Emission Scanning Electron Microscopy (FESEM) (Figure S9b) confirmed that the NSM-0.7 particles showed minimal agglomeration after 500 ORR test cycles compared to the initial sample. X-ray Diffraction (XRD) analysis (Figure S9c) revealed that after 500 cycles, the catalyst's crystallinity had decreased, but its phase remained unchanged. An additional hump observed between 2θ=20° and 30° is attributed to the presence of Vulcan carbon, which was mixed with the NSM material during catalyst preparation. This combined evidence confirms the excellent long-term stability of the NSM-0.7 nanoparticles.

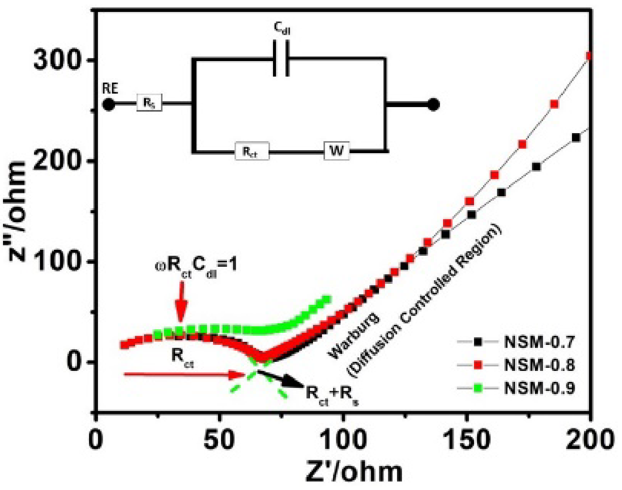

Figure S10. Impedance study of NSM perovskite oxides using EIS

Electrochemical Impedance Spectroscopy (EIS) is a powerful method used to study charge transfer dynamics and diffusion processes at the electrode-electrolyte interface. The Nyquist plots in Figure S10 illustrate the results for the synthesized electrocatalysts. Each graph is composed of a semicircle in the high-frequency spectrum and a linear section in the low-frequency spectrum. The semicircle's diameter signifies the charge-transfer resistance (R_{ct}), while the linear section's gradient mirrors the electrolyte's diffusion properties at the electrode's interface. The inset of Figure S10 displays an equivalent circuit schematic used to analyse the experimental data. This circuit consists of: Rs: Resistance of the solution, W: Warburg impedance, which demonstrates diffusion, R_{ct}: Charge-transfer resistance, and CPE: Constant phase element, which accounts for non-ideal capacitance [42]. The R_{ct} values for NSM-0.7, NSM-0.8, and NSM-0.9 were determined to be 53.13 Ω, 54.42 Ω, and 53.13 Ω, respectively.
